# Evaluating the Improvement in Shear Wave Speed Image Quality Using Multidimensional Directional Filters in the Presence of Reflection Artifacts

**DOI:** 10.1109/TUFFC.2016.2558662

**Published:** 2016-04-27

**Authors:** Samantha L. Lipman, Ned C. Rouze, Mark L. Palmeri, Kathryn R. Nightingale

**Affiliations:** Duke University, Durham, NC 27708 USA

**Keywords:** 2-D shear wave speed, 3-D shear wave elastography, 3-D shear wave speed, 3-D stiffness reconstruction, acoustic radiation force, acoustics, biomechanics, biomedical ultrasonics, directional filter, elasticity, imaging, propagation, shear wave elasticity imaging, shear wave propagation, simulations, ultrasonic imaging

## Abstract

Shear waves propagating through interfaces where there is a change in stiffness cause reflected waves that can lead to artifacts in shear wave speed (SWS) reconstructions. Two-dimensional (2-D) directional filters are commonly used to reduce in-plane reflected waves; however, SWS artifacts arise from both in- and out-of-imaging-plane reflected waves. Herein, we introduce 3-D shear wave reconstruction methods as an extension of the previous 2-D estimation methods and quantify the reduction in image artifacts through the use of volumetric SWS monitoring and 4-D-directional filters. A Gaussian acoustic radiation force impulse excitation was simulated in phantoms with Young’s modulus (*E*) of 3 kPa and a 5-mm spherical lesion with *E* = 6, 12, or 18.75 kPa. The 2-D-, 3-D-, and 4-D-directional filters were applied to the displacement profiles to reduce in-and out-of-plane reflected wave artifacts. Contrast-to-noise ratio and SWS bias within the lesion were calculated for each reconstructed SWS image to evaluate the image quality. For 2-D SWS image reconstructions, the 3-D-directional filters showed greater improvements in image quality than the 2-D filters, and the 4-D-directional filters showed marginal improvement over the 3-D filters. Although 4-D-directional filters can further reduce the impact of large magnitude out-of-plane reflection artifacts in SWS images, computational overhead and transducer costs to acquire 3-D data may outweigh the modest improvements in image quality. The 4-D-directional filters have the largest impact in reducing reflection artifacts in 3-D SWS volumes.

## I. Introduction

SHEAR wave elasticity imaging (SWEI) has been developed as a noninvasive, quantitative imaging technique that measures the mechanical properties of tissue [1]. SWEI uses a diagnostic ultrasound transducer to create an acoustic radiation force impulse (ARFI) excitation that induces micrometer-scale displacements [2]–[5]. This impulsive excitation generates shear waves that propagate orthogonally outward in all directions from the region of excitation. Typical transducers used for these imaging methods capture one imaging plane of this dynamic response. Current SWEI algorithms are limited to 2-D regions of interest (ROIs) and assume tissue homogeneity in small reconstruction kernels that estimate shear wave speed (SWS) using time-of-flight (TOF)-based algorithms [3]–[7]. Reconstructing an SWS image from these SWEI algorithms in heterogeneous tissues can introduce a variety of artifacts, including SWS underestimation from reflections at stiffness interfaces [8], [9].

Applying a 2-D-directional filter in Fourier (*k_x_*, *ω*) space has been shown to reduce artifacts from the reflected waves [10] and to separate leftward from rightward traveling waves as used in comb-push ultrasound shear elastography [11], [12]. Three-dimensional-directional filters have also been used to separate the waves from multiple simultaneous steered excitations, for shear compounding [13]. However, for structures such as a spherical inclusion, these reflected waves come from both in-plane and out-of-plane stiffness interfaces. Recently, Gennisson *et al.* [14] performed 4-D shear wave imaging using multiple excitations and a cylindrical directional filter to create volumetric SWS maps in phantoms and healthy breast tissue. These methods required two coordinate transforms, and filtered each depth independently for diverging waves. In this paper, we implement 2-D, 3-D, and 4-D Cartesian directional filters and compare their performance in creating SWS images to those created without directional filtering. We also extend the robust 2-D shear wave reconstruction methods, as described in [13], to a 3-D shear wave reconstruction method and compare the results of 2-D reconstructions to their 3-D counterparts.

## II. Methods

The standard conventions for ultrasonic imaging principle axes were used in this paper, where *z* refers to depth, *x* to lateral position, and *y* to elevation position.

### A. Simulation Methods

Previously validated finite-element (FE) models were used to simulate the dynamic response of an elastic solid to impulsive, acoustic radiation force excitations [15]. These models simulate the diffraction pattern of a particular imaging case and the applied point loads are calculated from

(1) $$\vec{F}=\frac{2\alpha \vec{I}}{c}$$

where $\vec{F}$ is the force per unit volume, *α* is the absorption coefficient, $\vec{I}$ is the intensity of the acoustic beam, and *c* is the speed of sound in the medium.

In this paper, the spatial distribution of acoustic radiation force was modeled by 3-D Gaussian distributions (2), as shown at the bottom of this page, which are readily modified for studying the impact of excitation geometry on SWEI (Table I), where ∏((*t*/*T*_ON_)−(1/2)) is a rect function that sets the pulse duration to start at *t* = 0 and end at *t* = *T*_ON_. Three different excitations were simulated for imaging each phantom: 1) 1:1 *x*–*y* aspect ratio with a full-width at half-maximum (FWHM) of 0.5 mm in *x* and *y*; 2) 1:4 *x*–*y* aspect ratio with an FWHM of 0.5 mm in *x* and 2 mm in *y*; and 3) 1:1 *x*–*y* aspect ratio with an **FWHM** of 1 mm in *x* and *y* (Table I). The peak amplitude, *A*, was chosen for each excitation to create maximum displacements similar to those seen experimentally (10–15 *μ*m).

Excitations 1 and 2 were used to demonstrate the impact of the directional filters and compare 2-D and 3-D SWS estimation algorithms. Excitation 3 was used to demonstrate these filtering and SWS estimation methodologies with geometries more realistic for current hardware. The parameters of Excitation 3 were calculated to create a similar focal configuration to the piston used to generate an ARFI excitation in the previous 3-D SWEI experiments [16], [17], by setting the standard deviations (*σ*) of the 3-D Gaussian (2), such that the FWHM of the Gaussian excitation force in each dimension would be similar to the beamwidths of the piston’s ARFI excitation.

The 3-D, dynamic response of a linear elastic solid to this excitation was solved through the balance of linear momentum using LS-DYNA3D (Livermore Software Technology Corp., Livermore, CA) with an explicit, time-domain method. The FE mesh had the dimensions of 20×10×40 mm^3^ with uniform 0.1 mm nodal spacing. The model used nonreflecting boundaries on the outer *x*–*z* and *y*–*z* faces to simulate a semi-infinite volume without reflection artifacts at the edges of the mesh. The top and bottom boundaries of the mesh were fixed to prevent the bulk motion of the model from the excitation. The symmetric nature of an ARFI excitation about the axial–lateral plane allowed the simplification of the model to half symmetry to reduce the memory and computational runtime. Simulations were performed for a total time of 12 ms with intermediate results saved at the intervals of 100 *μ*s. Calculations were performed on a Linux cluster with an average CPU speed of 2.6 GHz.

(2) $$F(x,y,z,t)=A\;\Pi \left(\frac{t}{T_{\text{ON}}}-\frac{1}{2}\right)\exp\left(-\left(\frac{{\left(x-x_{0}\right)}^{2}}{2\sigma_{x}^{2}}+\frac{{\left(y-y_{0}\right)}^{2}}{2\sigma_{y}^{2}}+\frac{{\left(z-z_{0}\right)}^{2}}{2\sigma_{z}^{2}}\right)\right)\hat{z}$$

The *z*-displacements through time were extracted over the entire extent of the mesh. The resulting displacements were reflected across the axial–lateral (*y* = 0) symmetry plane, to create a full field of view for processing and analysis. The *x*- and *y*-displacements were not considered for this paper, because the ability to track micrometer-scale displacements ultrasonically is superior in the axial dimension.

The dynamic response of each excitation was simulated in three phantoms with a spherical inclusion with a 5 mm diameter centered at the focal depth of 21 and 5 mm laterally offset from the push axis, with Young’s moduli (*E*) of 6, 12, or 18.75 kPa in a 3 kPa background medium. These different stiffness contrasts modulate the amplitude of the reflected waves by increasing the shear impedance mismatch from the lesion-background interfaces [18]. An expected SWS (*c_T_*) for each material was calculated based on

(3) $$c_{T}=\sqrt{\frac{E}{2\rho (1+\nu )}}$$

with density *ρ* = 1g/cm^3^, and Poisson’s ratio *ν* = 0.495 (nearly incompressible).

### B. Directional-Filtering Methods

The 4-D volume (*x*, *y*, *z*, and *t*) of *z*-displacements was fast Fourier transformed using the MATLAB (Mathworks R2014b, Natick, MA) built-in function fftn. The dimensions of the directional filters in the spatio-temporal domain and the frequency domain are listed in Table II. The dimensions of each filter refer to the temporal dimension with the remaining dimensions referring to spatial dimensions. For example, the 4-D-directional filter refers to three dimensions of space and time, the 3-D-directional filter to two dimensions of space and time, and so on. The 4-D-directional filter is comprised of two separable filters: one varies with the three spatial frequencies, *k_x_*, *k_y_*, and *k_z_*, and the other varies with the spatial frequencies in *k_x_* and the temporal frequencies, *ω*. The first component of the 4-D-directional filter is an extension to three dimensions of the directional filters used in [13] and [19]. This filter weighs the spatial frequency spectra based on a cosine raised to a power (*q*) about the assumed direction of shear wave propagation. The magnitude of this filter was calculated from

(4) $$\cos\theta \frac{\vec{u}\cdot \vec{k}}{\left|\vec{u}||\vec{k}\right|}$$

and

(5) $$r=\cos^{q}\theta$$

where $\vec{u}$ is the assumed direction of propagation, and $\vec{k}$ is each point in *k*-space.

The center of the excitation and the center of the lesion are in the *y* = 0 plane. To reconstruct an SWS image in that plane, the shear wave is assumed to propagate is the +*x*-direction, and the 4-D-directional filters are implemented with $\vec{u}=\{1,0,0\}$. Typical values of *q* used in the previous work are 2 or 3 [11]–[13], [19]. The simulations in this paper use a single ARFI excitation and are filtered for propagation in one direction to create a shear wave reconstruction, so a value of *q* = 2 was chosen to create a broader angular width of the directional filter.

**Fig. 1:**
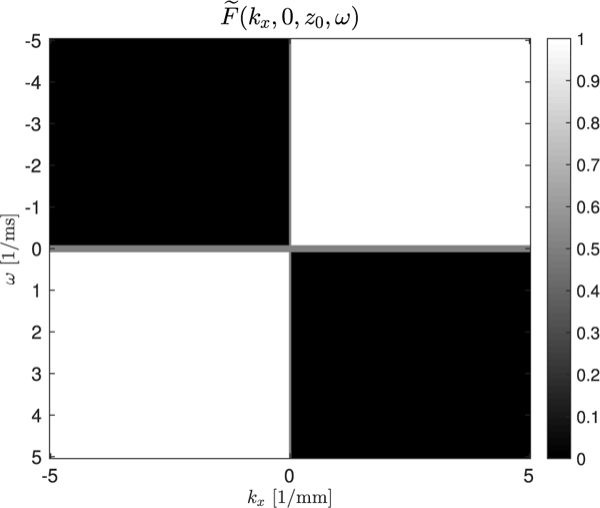
Two-dimensional (*k_x_*, *ω*) Fourier domain filter for the spatial frequencies (*k_x_*) and temporal frequencies (*ω*) that select waves moving in the positive lateral direction.

**Fig. 2:**
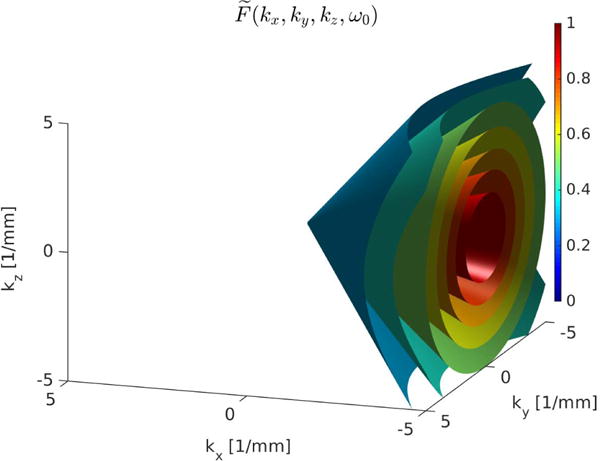
Magnitude of the 4-D Fourier domain directional filter that selects waves moving in the lateral (+*x*) direction shown for a single *ω*_0_. This filter was constructed from (4) and (5), $\vec{u}=\{1,0,0\}$ and *q* = 2. This filter attenuates the spatial frequencies that are not predominantly related to the waves moving in the +*x*-direction. Isosurfaces of the 4-D filter at *ω*_0_ from 0.3 to 0.9 in the increments of 0.1 to demonstrate the volumetric nature and symmetries of the filter.

The second component of the 4-D-directional filter is a space-time filter previously shown in [10] for the *k_x_*−*ω* dimensions, to select only the waves traveling in the positive lateral direction (Fig. 1). The boundaries of the quadrants were set to a value of 0.5 to avoid ringing artifacts from sharp filter edges. Each filter was applied to the Fourier transformed *z*-displacement data and then inverse Fourier transformed using ifftn to return the filtered *z*-displacement fields. The distribution of the magnitude of these combined filters is shown for a single *ω*_0_ with an isosurface plot of points in the filter with the magnitude of at least 0.3 in Fig. 2.

**Fig. 3:**
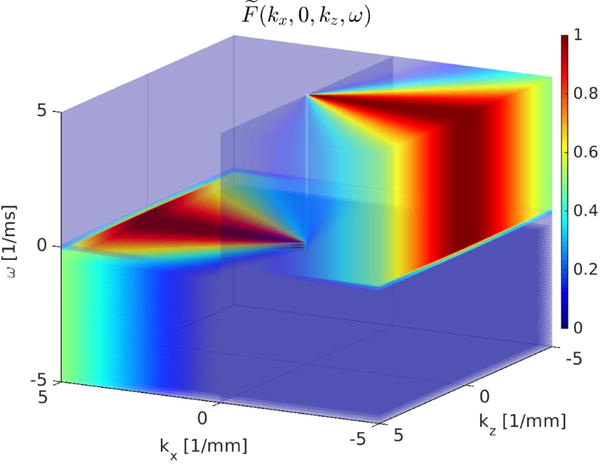
Magnitude of the 3-D Fourier domain directional filter that selects waves moving in the lateral (+*x*) direction. This filter was constructed from (4) and (5), $\vec{u}=\{1,0\}$ and *q* = 2. This filter attenuates the spatial frequencies that are not predominantly related to the waves moving in the +*x*-direction. As the values of this filter approach zero, they are shown with increased transparency.

The same formulation can be used to create 3-D-directional filters. The 3-D volume of the *y* = 0 plane (*x*, *z*, and *t*) is fast Fourier transformed, and the first filter computed in terms of *k_x_* and *k_z_*, where $\vec{u}=\{1,0\}$. The magnitude of this 3-D-directional filter is shown over the entire volume in Fig. 3. Transparency of the volume is increased as the values approach zero for better visualization. The 2-D-directional filter reported in [10] uses the same 3-D volume data, but each depth is individually Fourier transformed in 2-D, and the filter shown in Fig. 1 is applied.

### C. Shear Wave Speed Estimation Methods

To distinguish the artifacts from the reflected waves from localized changes in the direction of shear wave propagation, SWS images were reconstructed using the cross-correlation-based methods described in [13] and [20]. For each pair of shear wave signals, the entire displacement through time profiles were cross correlated, and parabolic subsample estimation was used to determine the time lag (Δ*t*) between the signals using

(6) $$\Delta t=[\underset{j}{\text{argmax}}\;CC(j)]/\text{PRF}$$

where PRF is the sampling rate through slow time.

The velocity of the pixel at the center of the patch can be calculated from

(7) $$V(m,n)=\frac{p\cdot \Delta x}{\Delta t}$$

where Δ*x* is the spatial resolution of the pixels, and *p* is the size of the patch in pixels. A patch of *p* = 6 was used in these studies. The component *V_x_* can be computed using (8), as shown at the bottom of this page, as a sum weighted by the normalized correlation coefficients (CC*_x_*) and the reciprocal of the distance (*r*) computed from

(8) $$V_{x}(m,n)=\sum_{i=m-h\prime }^{m+h\prime }\sum_{j=n-h\prime }^{n+h\prime }\left\{V_{x}(i,j)\cdot \frac{CC_{x}{\left(i,j\right)}^{2}/r(i,j)}{\sum_{i=m-h\prime }^{i=m+h\prime }\sum_{j=n-h\prime }^{j=n+h\prime }CC_{x}{\left(i,j\right)}^{2}/r(i,j)}\right\},\;h\prime =\frac{w-p}{2}$$

(9) $$r(i,j)=\left\{ \begin{array}{l} 1,\\ \sqrt{{\left(i-m\right)}^{2}+{\left(j-n\right)}^{2},} \end{array}\right.\;\begin{array}{l} i=m\;\text{and}\;j=n\\ \text{else} \end{array}$$

to the center pixel of the 2-D window (*w*) [13]. A window size of *w* = 10 was used in these studies. The component *V_z_* can be computed with a similar equation, substituting *V_z_* for *V_x_* and CC*_z_* for CC*_x_*.

The 2-D wave speed can be computed from

(10) $$V_{2-D}=\frac{V_{x}V_{z}}{\sqrt{V_{x}^{2}+V_{z}^{2}}}$$

using the weighted sums of *V_x_* and *V_z_* [13].

(11) $$V_{x}(l,m,n)=\sum_{i=l-h\prime }^{l+h\prime }\sum_{j=m-h\prime }^{m+h\prime }\sum_{k=n-h\prime }^{n+h\prime }\left\{V_{x}(i,j,k)\cdot \frac{CC_{x}{\left(i,j,k\right)}^{2}/r(i,j,k)}{\sum_{i=l-h\prime }^{i=l+h\prime }\sum_{j=m-h\prime }^{j=m+h\prime }\sum_{k=n-h\prime }^{k=n+h\prime }CC_{x}{\left(i,j,k\right)}^{2}/r(i,j,k)}\right\},\;h\prime =\frac{w-p}{2}$$

(12) $$r(i,j,k)=\left\{ \begin{array}{l} 1\\ \sqrt{{\left(i-l\right)}^{2}+{\left(j-m\right)}^{2}+{\left(k-n\right)}^{2}} \end{array}\right.\;\begin{array}{l} i=l,\;j=m,\;\text{and}\;k=n\\ \text{else} \end{array}$$

**Fig. 4:**
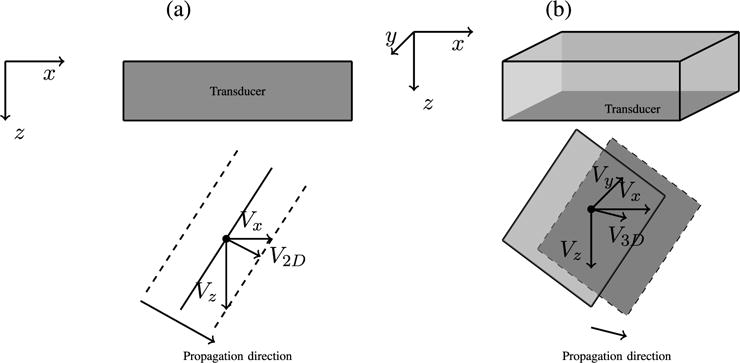
(a) Normalized cross-correlation methods to determine SWSs using a 1-D finite window in the assumed direction of propagation will overestimate the SWS by measuring *V_x_* as the SWS, rather than *V*_2-D_, as described in [13]. If both *V_x_* and *V_z_* are measured, then the true shear wave velocity (*V*_2-D_) can be reconstructed for a wave propagating in any direction within the imaging plane. (b) Equations (12) and (13) extend the method to include the third dimension (*V_y_*) to capture the true velocity (*V*_3-D_) of a wave propagating in any direction within the volume.

It has been shown that the waves traveling in and out of the imaging plane can lead to bias in shear wave reconstructions [21]. To take advantage of the full 4-D data set, these cross-correlation methods were extended to 3-D to create volumetric SWS reconstructions (Fig. 4). The same SWS estimation parameters (*p* = 6 and *w* = 10) were used for both the 2-D and 3-D reconstructions. An example of the 3-D summation equation is shown for *V_x_* in (11), as shown at the bottom of this page, where the velocity estimates are weighted based on correlation coefficient and reciprocal distance from the center pixel of a 3-D volume (12), as shown at the bottom of this page. Similar equations are used to compute *V_y_* and *V_z_*. The 3-D SWS is then calculated from

(13) $$V_{3-D}=\frac{V_{x}V_{y}V_{z}}{\sqrt{V_{x}^{2}V_{y}^{2}+V_{x}^{2}V_{z}^{2}+V_{y}^{2}V_{z}^{2}}}$$

where *V_x_*, *V_y_*, and *V_z_* are the weighted sums of each component of the velocity.

### D. Analysis Methods

The axial extent of each data set was truncated to depths between 1 and 3 cm to reduce the memory requirements of 4-D processing. White Gaussian noise with amplitude creating a 25-dB signal-to-noise ratio (SNR) was added to the 4-D *z*-displacements using the MATLAB built-in function awgn to simulate the jitter of tracked displacements without the computational complexity of simulating volumetric tracking [22]. Ten noise realizations were simulated for each set of imaging parameters. The displacement volumes with added noise were then 4-D-directionally filtered; the *y* = 0 imaging plane of the unfiltered data sets was also used to perform SWS reconstruction without directional filtering, in addition to 2-D- and 3-D-directional filtering. The *k*-space dimensions for each type of directional filter are listed in Table II. Although the simulations adequately capture the dynamic response with a PRF of 10 kHz, the displacement profiles were upsampled to 50 kHz using spline interpolation for all reconstructions to achieve finer temporal resolution [23]. The 2-D- and 3-D-filtered data sets, as well as the *y* = 0 plane of the unfiltered and 4-D-filtered data sets, were used to create 2-D SWS images. In addition, the entire unfiltered and 4-D-filtered volumes were used to reconstruct 3-D SWS image volumes.

A 2-D 0.5 × 0.5 mm^2^ median filter was applied to reduce outlier pixels in the 2-D SWS reconstructions, and a 3-D 0.5×0.5×0.5 mm^3^ median filter was applied to reduce outlier pixels in the 3-D SWS volume. Image quality was evaluated by computing contrast-to-noise ratio (CNR) for each SWS image from

(14) $$\text{CNR}=\frac{S_{i}-S_{o}}{\sqrt{\sigma_{i}^{2}+\sigma_{o}^{2}}}$$

using a circular ROI with a 4 mm diameter within the lesion and two half circles with the same diameter above and below the lesion [24]. The regions used to calculate this metric are shown in black dashed outlines and the true location of the lesion is indicated by the magenta dashed–dotted circle overlaid on each image (Figs. 6–9, 12, and 13). Given the gradients and heterogeneity around the edges of each lesion, regardless of filtering method, the analysis was restricted to a smaller region half of the window length from the lesion edges, so the metrics of CNR and bias would not be strongly influenced by the edge effects. The accuracy of each SWS image was assessed by computing percent bias using

(15) $$\%\text{Bias}=100\times \frac{\hat{c_{T}}-c_{T}}{c_{T}}$$

at each pixel within the lesion using the same 4-mm-diameter ROI used to calculate CNR, where $\hat{c_{T}}$ is the reconstructed SWS and *c_T_* is the true SWS calculated from (3) using the input material properties. The data from Excitation 3 were used to study the effects of sparse spatial sampling on directional filtering and SWS estimations. The displacement fields for these imaging cases were downsampled to 0.3 mm isotropic spacing, and the absolute size of the kernels was increased for 2-D and 3-D SWS reconstructions (*p* = 4 and *w* = 8). Median filters of 1.5 × 1.5 mm^2^ (2-D) and 1.5 × 1.5 × 1.5 mm^3^ (3-D) were applied to their respective data sets.

## III. Results

**Fig. 5:**
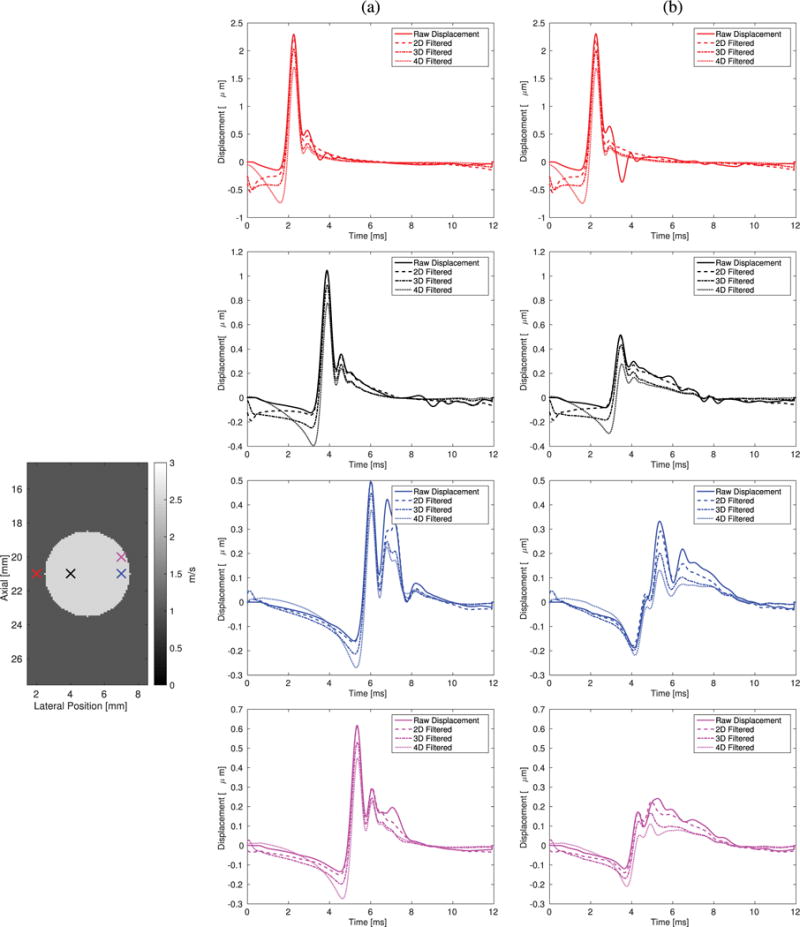
Raw displacement profiles using Excitation 1 (Table I) at four locations without and with 2-D-, 3-D-, and 4-D-directional filtering. The color of the displacement profiles corresponds to the position of the same color marker in the phantom on the left. The red, blue, and black markers are at a depth of 21 mm and the magenta marker is at a depth of 20 mm in (a) 6 and (b) 18.75 kPa lesions. Directional filtering increases the negative displacements that precede the initial wavefront, which contributes to more accurate SWS reconstructions. Top row: displacement profiles outside the lesion have the same amplitude, and directional filtering suppresses ringing later in time. Second row: within the lesion, avoiding areas of artifact (black), the stiffer lesion has lower peak displacement amplitude and a higher relative reflected wave amplitude, indicating a lower SNR compared with the softer lesion. Third row: in the 6 kPa lesion, the reflected wave is separated from the initial wavefront, while in the 18.75 kPa lesion, the magnitude of the reflected waves exceeds the magnitude of the initial wavefront, which leads to SWS overestimation. Bottom row: in the 6 kPa lesion, the reflected wave is separated from the initial wavefront and is smaller in magnitude, while in the 18.75 kPa lesion, the magnitude of the reflected waves exceeds that of the initial wavefront causing SWS underestimation. In all cases, directional filtering decreases the reflected wave energy.

Raw displacement profiles from the simulations using Excitation 1 (1:1 *x*–*y* aspect ratio and 0.5 mm FWHM) in the 6 and 18.75 kPa lesions are shown in Fig. 5, along with the 2-D-, 3-D-, and 4-D-directionally filtered data from these cases without added noise. Four locations are shown: 1) outside the lesion; 2) inside the lesion in an area without reconstruction artifact; 3) in the areas of overestimation; and 4) in the areas of underestimation due to reflected wave dynamics. These profiles demonstrate the relative magnitude and morphology of the reflected shear waves as a function of lesion material contrast.

**Fig. 6:**
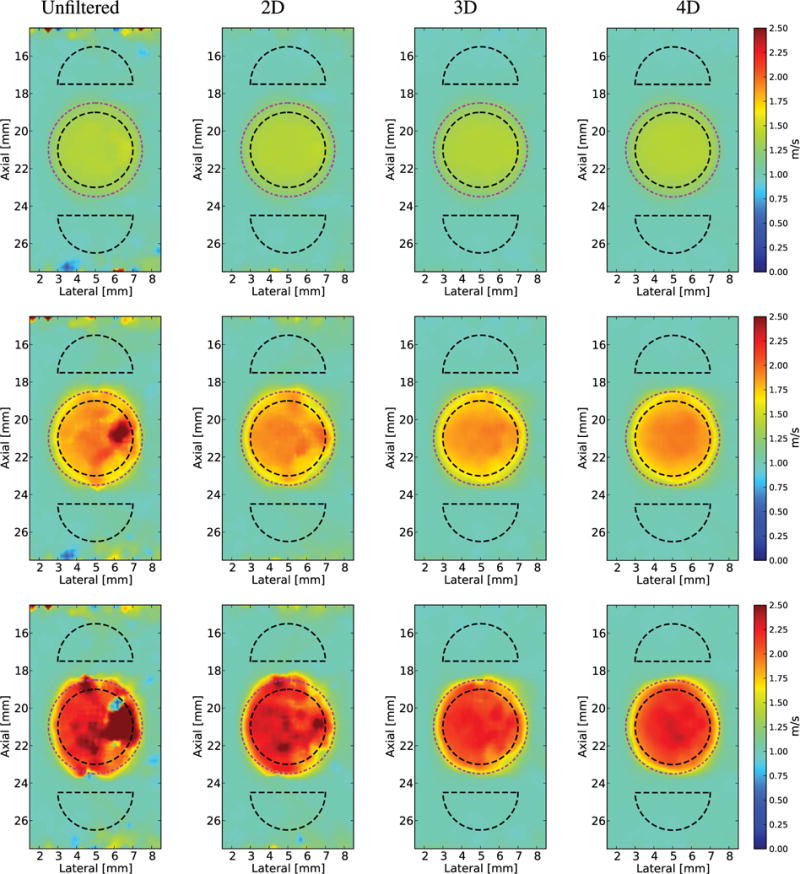
Reconstructed 2-D SWS (*V*_2-D_) images of a spherical lesion in an *E* = 3 kPa and *c_T_* = 1 m/s background using Excitation 1 (1:1 *x*–*y* aspect ratio, Table I) with increased dimensions of filtering in each column from left to right. Black dashed outlines indicate the ROIs used for CNR computation and the true location of the lesion is indicated by the magenta dashed–dotted circles. In all cases, increased dimensions of filtering reduced the noise. Top row: *E* = 6 kPa and *c_T_* = 1.4 m/s lesion. This is the smallest stiffness ratio, and the reflected waves are not severe enough to introduce a reflection artifact. Middle row: *E* = 12 kPa and *c_T_* = 2 m/s lesion. Increasing the dimensions of filtering decreases the overestimation/underestimation of the SWS on the right edge of the lesion. Bottom row: *E* = 18.75 kPa and *c_T_* = 2.5 m/s lesion. Increasing the dimensions of filtering decreases the overestimation/underestimation of the SWS on the right edge of the lesion.

**Fig. 7:**
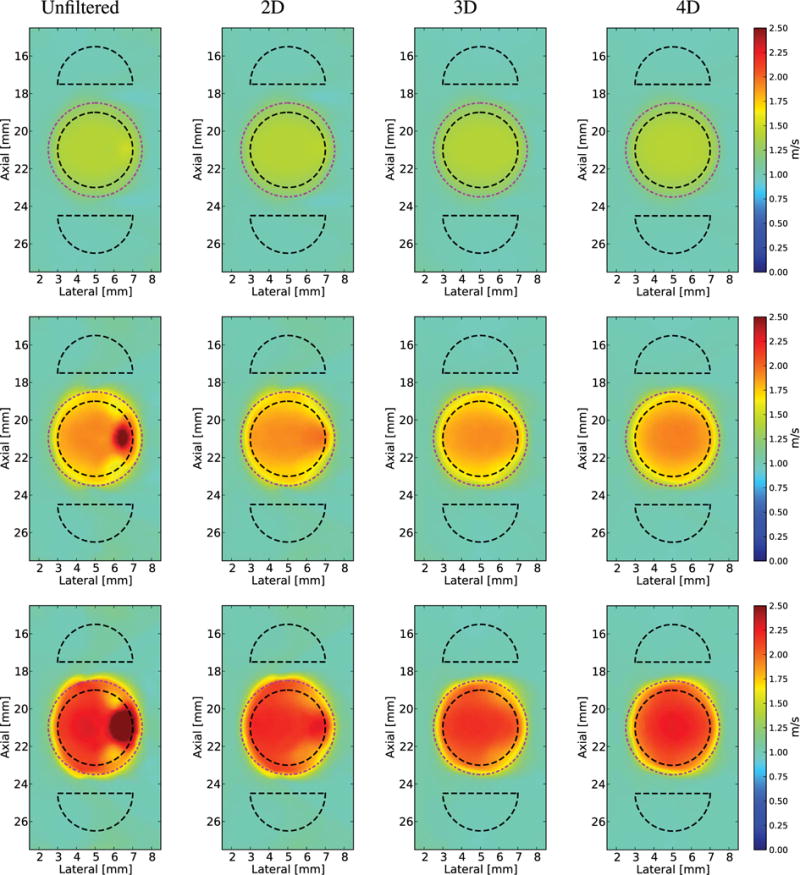
Reconstructed 2-D SWS (*V*_2-D_) images of a spherical lesion in an *E* = 3 kPa and *c_T_* = 1 m/s background using Excitation 2 (1:4 *x*–*y* aspect ratio, Table I) with increased dimensions of filtering in each column from left to right. Black dashed outlines indicate the ROIs used for CNR computation and the true location of the lesion is indicated by the magenta dashed–dotted circles. In all cases, increased dimensions of filtering reduced the noise. Top row: *E* = 6 kPa and *c_T_* = 1.4 m/s lesion. This is the smallest stiffness ratio, and the reflected waves cause a very small area of overestimation in SWS that is removed by the 2-D-directional filter. Middle row: *E* = 12 kPa and *c_T_* = 2 m/s lesion. Increasing the dimensions of filtering decreases the overestimation/underestimation of the SWS on the right edge of the lesion. Bottom row: *E* = 18.75 kPa and *c_T_* = 2.5 m/s lesion. Increasing the dimensions of filtering decreases the overestimation/underestimation of the SWS on the right edge of the lesion.

The SWS images in Figs. 6 and 7 were reconstructed using 2-D SWS estimation (*V*_2-D_). The SWS images created from Excitation 1 are shown without directional filtering and after 2-D-, 3-D-, and 4-D-directional filtering from the phantoms with 6, 12, and 18.75 kPa spherical inclusions in Fig. 6. The unfiltered SWS images from the 12 and 18.75 kPa lesions show the regions of both SWS underestimation and overestimation at the right edge of the lesion due to the detection of reflected waves. This artifact, along with the noise of the image, is mitigated with the increasing of directional-filtering dimensionality. The reconstructed SWS images from the phantom with a 6 kPa spherical inclusion did not exhibit SWS overestimation or underestimation at the right boundary of the lesion due to the relatively low amplitude of the reflected waves [Fig. 5 (left column)]. This set of conditions was included to show that the higher levels of filtering do not cause corruption in an SWS image without significant reflection artifacts.

To demonstrate the impact of the excitation geometry on both the shear wave reflection artifacts and the ability of directional filtering to remove this artifact, Excitation 2 (1:4 *x*–*y* aspect ratio, and 0.5 and 2 mm FWHM, respectively) was used to image the same phantoms, and SWS images were reconstructed without directional filtering and after 2-D-, 3-D-, and 4-D-directional filtering (Fig. 7).

**Fig. 8:**
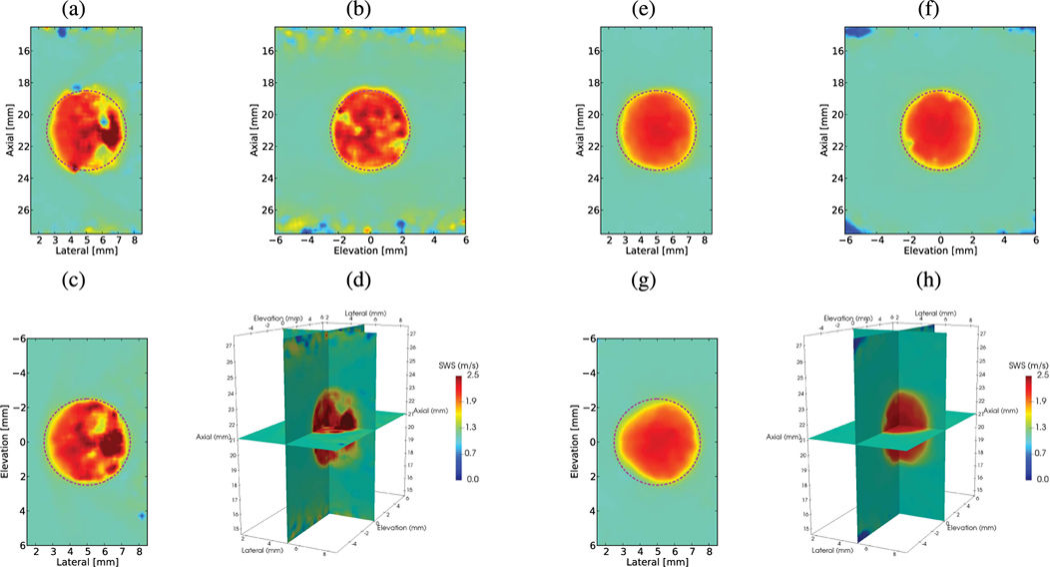
Orthogonal planes through the center of the lesion in a reconstructed 3-D SWS volume (*V*_3-D_) from a simulation using Excitation 1 in an 18.75 kPa and *c_T_* = 2.5 m/s lesion without directional filtering and after 4-D-directional filtering. (a) Axial–lateral plane (*y* = 0 mm). (b) Axial–elevation plane (*x* = 5 mm). (c) Lateral–elevation plane (*z* = 21 mm). (d) Orthogonal planes in 3-D space. (e) Axial–lateral plane (*y* = 0 mm). (f) Axial–elevation plane (*x* = 5 mm). (g) Lateral–elevation plane (*z* = 21 mm). (h) Orthogonal planes in 3-D space. The true location of the lesion is indicated by the magenta dashed–dotted circles. Note the reflection artifacts, which appear in the volume, seen at the right edge of the lesion in (a) and (c), but also in the jagged edge of the lesion in (b). Note the distortion of the lesion in (c), elevation plane at the edge closest to the source of the excitation an effect that is not changed by directional filtering (g).

**Fig. 9:**
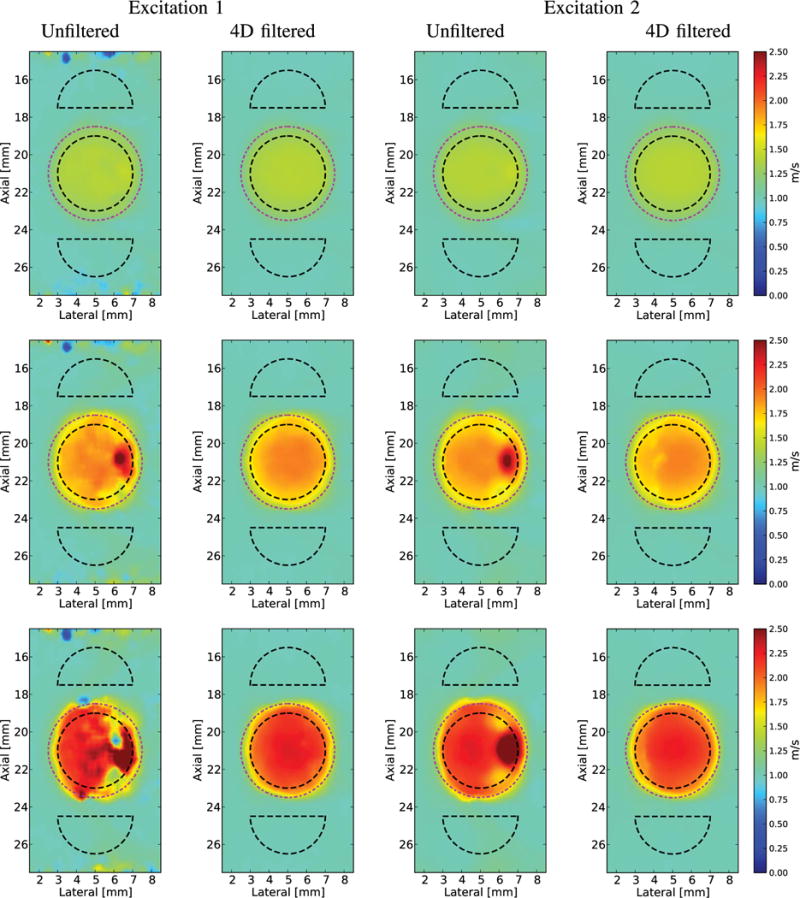
Axial–lateral (*y* = 0) images from a reconstructed 3-D SWS (*V*_3-D_) volume of a spherical lesion in an *E* = 3 kPa and *c_T_* = 1 m/s background using Excitation 1 (Table I) in the two leftmost columns and Excitation 2 in the rightmost two columns. The left column for each excitation shows a plane of the reconstruction without directional filtering and the right column after 4-D filtering. Black dashed outlines indicate the ROIs used for CNR computation and the true location of the lesion is indicated by the magenta dashed–dotted circles. In all cases, increased dimensions of filtering reduced the noise. Top row: *E* = 6 kPa and *c_T_* = 1.4 m/s lesion. This is the smallest stiffness ratio, and the reflected waves are not severe enough to introduce a significant reflection artifact. Middle row: *E* = 12 kPa and *c_T_* = 2 m/s lesion. Bottom row: *E* = 18.75 kPa and *c_T_* = 2.5 m/s lesion. Increasing the dimensions of the SWS estimator alone is not enough to reduce the reflection artifact, but the directional filtering removes the overestimation/underestimation of the SWS on the right edge of the lesion for the two stiffest lesions.

In Fig. 8, the reconstructed SWS volumes are shown using three orthogonal planes through the center of the lesion for Excitation 1 in the phantom with an 18.75 kPa spherical inclusion without directional filtering and after 4-D-directional filtering. Fig. 9 shows the axial-lateral plane at *y* = 0 of the *V*_3-D_ SWS volumetric reconstructions using Excitations 1 and 2.

**Fig. 10:**
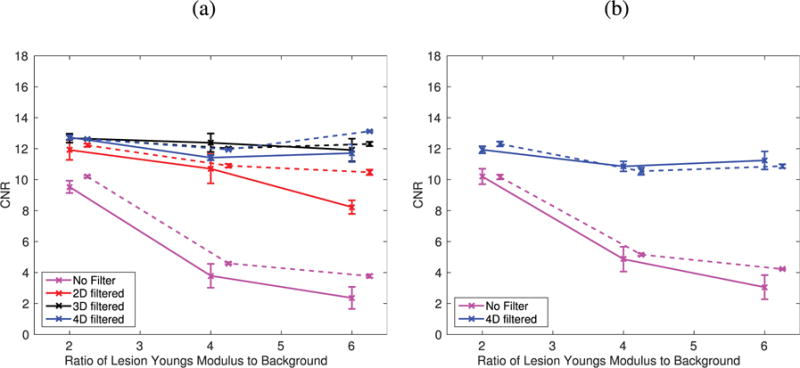
CNR for the imaging cases shown in Figs. 6, 7, and 9 using ten noise realizations. Calculated values from SWS images generated from Excitation 1 (Table I) are shown with solid lines, while the values generated from Excitation 2 are indicated with dashed lines. The lesion stiffnesses used were the same for both excitations. (a) CNR from 2-D SWS reconstructions (*V*_2-D_) from Figs. 6 and 7. Note that the CNR increases with increased filtering dimension for each lesion regardless of stiffness ratio. (b) CNR from *y* = 0 plane of 3-D SWS reconstructions (*V*_3-D_) using ROIs as shown in Fig. 9 with black dashed lines. Note that the CNR calculated from the (*V*_3-D_) 4-D-filtered images is similar to the CNR values calculated from the 2-D reconstructions (*V*_2-D_), but the CNR calculated from the unfiltered 3-D reconstructions (*V*_3-D_) is higher than the CNR calculated from the unfiltered 2-D reconstructions (*V*_2-D_), because the waves coming in and out of the imaging plane contribute to the artifact.

Image quality was evaluated by calculating CNR for each reconstructed 2-D SWS image [*V*_2-D_, Fig. 10(a)]. The equivalent CNR was calculated from the same plane (*y* = 0) for the 3-D SWS volumes [*V*_3-D_, Fig. 10(b)]. In these plots, the CNR values from images generated from an excitation with a 1:1 *x*–*y* aspect ratio are shown with solid lines and those generated from an excitation with a 1:4 *x*–*y* aspect ratio are shown with dashed lines. The lesion stiffnesses used were the same for both excitations, but the points from the 1:4 *x*–*y* aspect ratio have been offset for better visualization.

**Fig. 11:**
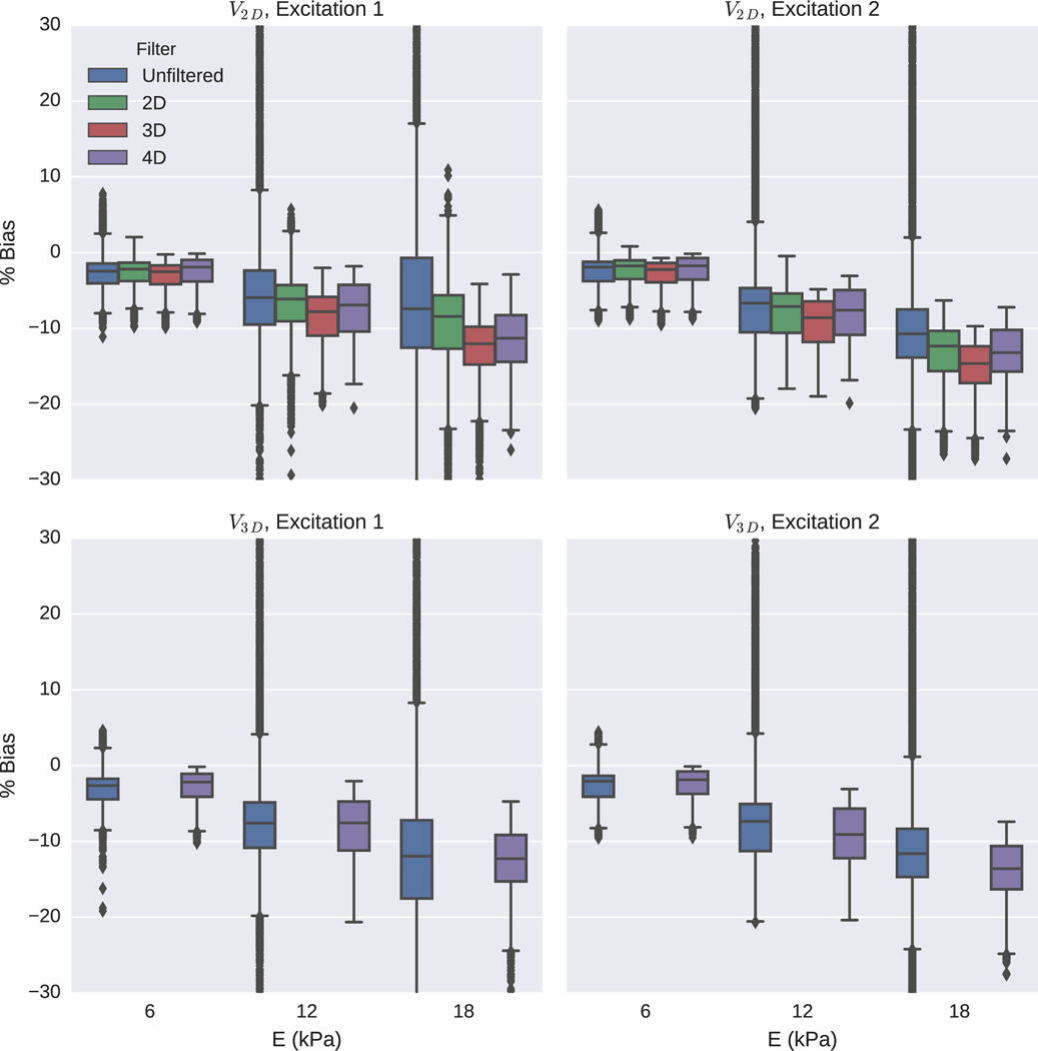
Percent bias of reconstructed SWS within the lesion is shown for each of the three lesion stiffnesses using Excitations 1 and 2, for both *V*_2-D_ and *V*_3-D_ SWS estimation. Outliers have been cut off over ±30% bias. Note that for each lesion, the median bias within the lesion does not significantly change with increased dimensionality of the directional filter, but the variance and number or outliers decrease. The variance of percent bias increases with stiffness as expected in SWEI imaging due to an increase in the shear wavelength from the higher mechanical contrast and increased noise in the higher SWS estimates from using a fixed reconstruction kernel [16], [25].

The accuracy of each reconstructed SWS image was evaluated by calculating a percent bias within the lesion using the same ROI used to calculate CNR (Figs. 6, 7, and 9). The distribution of the bias is shown in the box plots for each of the imaging and reconstruction cases (Fig. 11).

**Fig. 12:**
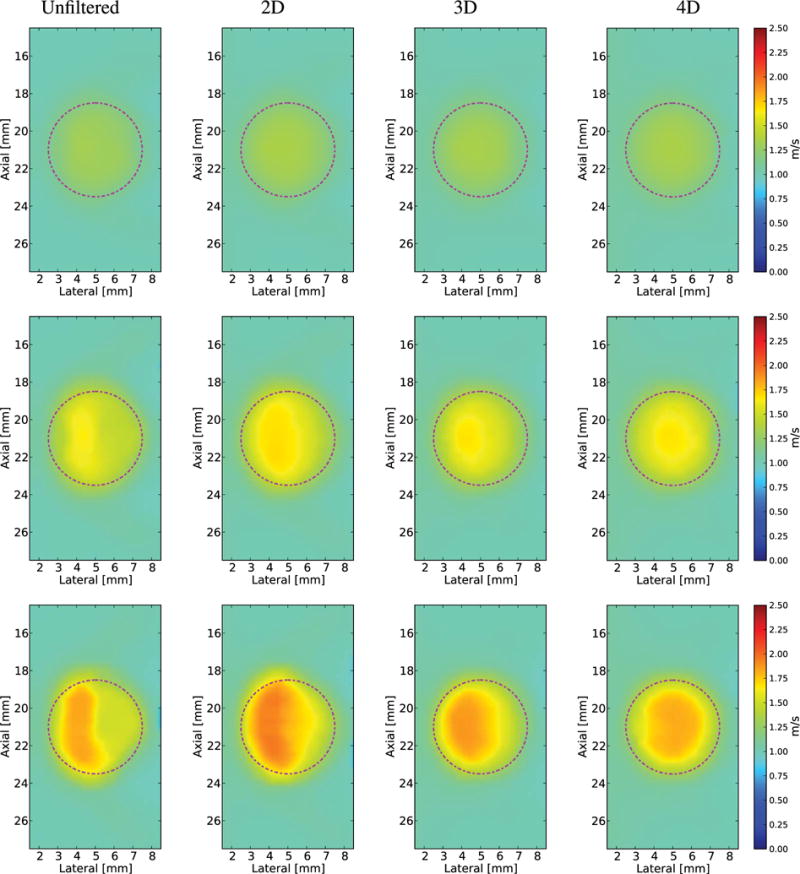
Reconstructed 2-D SWS (*V*_2-D_) images of a spherical lesion in an *E* = 3 kPa and *c_T_* = 1 m/s background using Excitation 3 (Table I) and decimated spatial sampling of 0.3 mm with increased dimensions of filtering in each column from left to right. The true location of the lesion is indicated by the magenta dashed–dotted circles. As with the more finely sampled cases in Fig. 6, increased dimensions of filtering reduced the noise. Top row: *E* = 6 kPa and *c_T_* = 1.4 m/s lesion. Middle row: *E* = 12 kPa and *c_T_* = 2 m/s lesion. Bottom row: *E* = 18.75 kPa and *c_T_* = 2.5 m/s lesion. For these three imaging cases, the reflection artifact appears as an underestimation on the right half of the lesion, the results in a gradient in reconstructed SWS across the lesion. Increasing the dimensionality of directional filtering creates a more uniform estimate within the lesion. Note that the directional filtering does not overcome the overall underestimation of SWS within the lesion in the two stiffest lesions that occurs due to the inability of the larger shear wavelength to resolve the correct speed in a structure of this size.

**Fig. 13:**
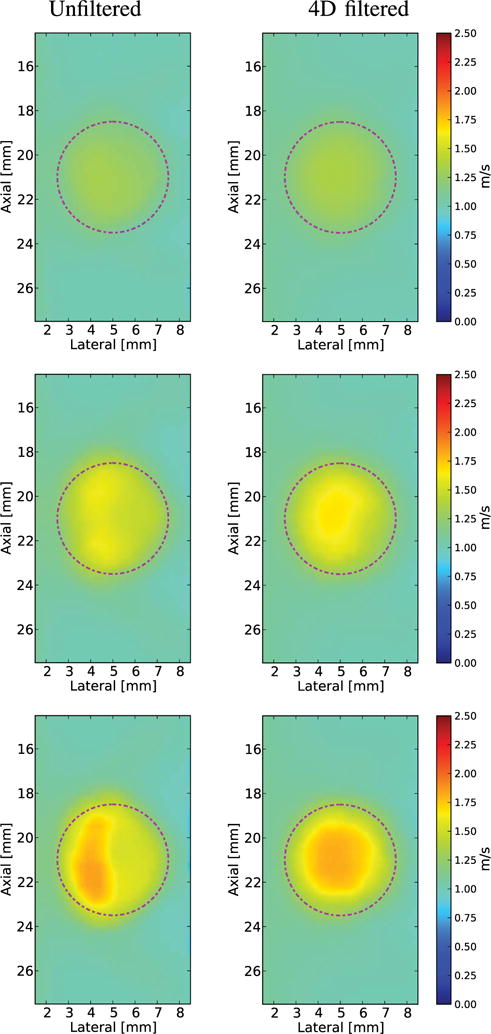
Axial–lateral (*y* = 0) images from a reconstructed 3-D SWS volume of a spherical lesion in an *E* = 3 kPa and *c_T_* = 1 m/s background using the decimated data of 0.3 mm spatial resolution for Excitation 3 (Table I). The left column shows a plane of the reconstruction without directional filtering and the right column after 4-D filtering. The true location of the lesion is indicated by the magenta dashed–dotted circles. Top row: *E* = 6 kPa and *c_T_* = 1.4 m/s lesion. Middle row: *E* = 12 kPa and *c_T_* = 2 m/s lesion. Bottom row: *E* = 18.75 kPa and *c_T_* = 2.5 m/s lesion. As with the *V*_2-D_ reconstructions in Fig. 12, the reflection artifact appears as an underestimation on the right half of the lesion, the results in a gradient in reconstructed SWS (*V*_3-D_) across the lesion. Increasing the dimensions of the SWS estimator alone is not enough to reduce the reflection artifact, but the directional filtering creates a more uniform estimate within the lesion. Note that the directional filtering does not overcome the overall underestimation of SWS within the lesion in the two stiffest lesions that occurs due to the inability of the larger shear wavelength to resolve the correct speed in a structure of this size.

The reconstructed SWS images (*V*_2-D_) using Excitation 3 (1:1 *x*–*y* aspect ratio and 1 mm FWHM) decimated to 0.3 mm spatial resolution are shown without directional filtering and after 2-D-, 3-D-, and 4-D-directional filtering from the phantoms with 6, 12, and 18.75 kPa spherical inclusions in Fig. 12. The unfiltered SWS images from the 12 and 18.75 kPa lesions show the regions of both SWS underestimation and overestimation at the right edge of the lesion due to the detection of reflected waves. This artifact is mitigated with each increase in dimensionality of directional filtering. The axial–lateral planes at *y* = 0 of the 3-D SWS volume reconstructions *(V*_3-D_) are shown in Fig. 13.

## IV. Discussion

The magnitude of shear wave reflections is a function of material contrast and location in structures, as demonstrated by the displacement profiles in Fig. 5. Directional filtering attenuates the energy in the displacement through time profiles, predominantly after the initial wavefront has propagated through a given region. In all locations and imaging configurations, directional filtering increases the negative displacements that precede the initial wavefront, and reflected wave energy is attenuated by all of the filters, with an increase in attenuation as a function of higher filter dimension.

The mechanical contrast of the relative inclusion stiffness compared with the background material dictates the reflected wave energy. As shown in Fig. 5, for an incident shear wave front with a maximum displacement of 10 *μ*m, the stiffer lesion (column B) exhibits smaller transmitted shear wave displacement than the more compliant lesion (column A), but the stiffer lesion creates greater relative reflected shear wave amplitude due to its higher shear impedance mismatch. The dependence of shear wave reflection energy on the materials being characterized impacts the relative improvements that the directional filters provide in each configuration.

Both qualitative and quantitative improvements in the reconstructed shear wave images can be seen by increasing the dimensionality of the applied directional filters. For low lesion stiffness contrast that generates little reflected wave artifact, increasing dimensionality of directional-filtering suppressed the broadband noise of the data, leading to very low bias and high CNR (Figs. 6, 7, 10, and 11). For increased lesion stiffness contrast, the artifactual stiff and soft appearance of the right boundary of the lesion is reduced with directional filtering, which increases the CNR of the lesion (Figs. 6, 7, and 10).

The shape of the shear wave source influences the accuracy of reconstructed SWS images. For the same material properties and spherical target, qualitative differences can be seen in the final reconstructed images made from Excitation 1 (Fig. 6) versus Excitation 2, which has a wider elevation dimension and is more consistent with commercial linear arrays (Fig. 7). Broader elevation source geometry causes a downshift in frequency content of the propagating shear wave in the lateral direction [26]. These longer shear wavelengths spatially smooth the reconstructed SWS image and reduce their spatial resolution. Longer shear wavelengths also increase the influence of reflected waves at boundaries with high mechanical contrast, since there is a longer time for the incident wave to interact with reflected wave (Fig. 7).

The shear wave SNR within the lesion varies between the different excitation geometries. Geometric spreading of the shear wave is greatest for Excitation 1, which best approximates a cylindrical wavefront that decays as $1/\sqrt{d}$, where *d* is the propagation distance. Rouze *et al.* [27] have shown that the excitations with different aspect ratios in *x* and *y* decay with a smaller fractional power rate, causing the shear wave SNR to be higher further from the source when imaging with broader elevation excitation sources.

Volumetric displacement fields enable 4-D-directional filtering and volumetric SWS estimation. Fig. 8 shows the SWS volumes reconstructed from Excitation 1, with and without 4-D-directional filtering in the stiffest lesion. The reflection artifact exists in three dimensions and the 4-D-directional filter is able to reduce this artifact within the volume. One limitation of the volumetric reconstruction methods is their sensitivity to the curvature of the wavefront at planes of symmetry. The methods detect positive speeds in one half-space and negative speeds in the other half-space due to the curvature of the wavefront and the fixed position of the signal relative to the reference. For example, at the *y* = 0 plane, there is greater cancellation of the positive estimates by the negative estimates from (11) resulting in an artificially low speed from the *V_y_* component from (13). The noise in this plane can be removed with volumetric median filtering.

For the excitation and lesion geometries studied, the improvements in CNR generally increase with increasing filter dimensionality, but with less improvement for increased dimensionality. This suggests that the shear wave reflections in the imaging plane have the greatest impact on image reconstruction, although out-of-plane reflected waves contribute to these artifacts with increasing significance, as the magnitude of the reflected waves increases with increasing material contrast. The 3-D filtering shows significant improvements in image quality over 2-D filtering, and because it can be performed on data obtained with a 1-D array at moderate computational cost, it would be beneficial to move to 3-D filtering in SWEI imaging.

Bias in SWS reconstruction was more significantly affected by excitation size than by directional filtering. For a given imaging configuration, the average bias of the reconstructed speeds within the lesion for varying filter configurations was affected by <15%; however, increasing the dimensionality of the directional filters increased the uniformity of the lesion images. The mean and the variance of percent bias increase with stiffness as expected in SWEI imaging due to an increase in the shear wavelength from the higher mechanical contrast and increased noise in the higher SWS estimates from using a fixed reconstruction kernel [16], [25].

Applying higher dimension filters did not corrupt SWS reconstructions, regardless of artifact from the reflected waves, so these filters could be used to improve SWS images without the *a priori* knowledge of the structures being imaged. This is important because the artifacts are dependent upon both lesion and beam geometries. The severity of the artifact is related to the size of lesion, since the curvature of the lesion acts as a mechanical lens that refocuses the reflected waves. The severity of this constructive interference due to refocusing is related to the shear wavelength, the ratio of the stiffness of the two materials, and the radius of curvature of the boundary. The ratios of the stiffness and shear wavelength are two parameters that have been varied in this paper and a radius of curvature was chosen to highlight this phenomena. In addition, the curved nature of both the lesion surface and the diverging wavefront leads to the shear wave interacting with the lesion, an oblique angle of incidence in elevation. The complex behavior leads to the focusing error seen on the left side of the lesion in Fig. 8(g).

The data sets simulated in this paper would require a matrix array for experimental implementation. Current matrix array transducers operate at lower frequencies that would lead to larger excitations and sparser spatial sampling of the displacement fields. Excitation 3 was simulated to approximate these more realistic experimental geometries [14], [16], [17]. These data were decimated to an isotropic spatial sampling of 0.3 mm, which is more typical of the spatial sampling that can be achieved with current imaging setups. The reflection artifact in images reconstructed from this excitation appears as a gradation of SWS within the lesion (Figs. 12 and 13). These image reconstructions show an increase in bias for the two stiffest lesions from the loss in resolution due to longer shear wavelength.

The SWS images with the highest CNR and decreased bias came from the processing using 4-D-directional filters; however, these filters use significant computational resources, requiring upward of 35 GB of RAM to perform the described processing in a single operation, whereas the 3-D-directional filtering required ~150 MB of RAM, and the 2-D filter required ~1 MB. These computational requirements can be reduced by taking advantage the separability of the filters and using parallel processing to reduce runtime.

The limitations of this paper include restricting the materials to linear elastic media. Soft tissues are known to be viscoelastic, and viscosity introduces frequency-based attenuation, which broadens the shear wave with propagation. This attenuation-based modulation of shear wave morphology could change the appearance of the reflection-based artifact as seen in the changes in the artifact appearance when using a larger excitation from Figs. 12 and 13. The directional filter and 3-D SWS reconstruction methods are dependent on adequate spatial and temporal sampling and should not be negatively impacted by viscosity, although that analysis is beyond the scope of this paper. It should also be noted that the duration of excitations used in this simulation study is short relative to many experimental studies, which are typically hundreds of microseconds. The initial displacements in our simulations were generated in a very soft (3 kPa) material. In linear, elastic materials, the frequency content of the excitation can change with excitation duration as the material stiffness increases; however, for this material, a change in duration should not affect the shear wave spectra [26]. A second limitation of this paper is the magnitude of displacements within the stiff inclusions (Fig. 5). The displacements within the lesion are <1 *μ*m, which is typically approaching the noise floor of ultrasonically tracked displacements. Experimentally, this would need to be overcome by using a longer, higher amplitude excitation to create a larger initial displacement, or several excitations would need to be used to synthesize an image. In practice, to perform 4-D-directional filtering and 3-D SWS estimation, a matrix array transducer is needed to generate 4-D displacement fields. Although currently 3-D volumetric imaging is limited by the availability, expense, and ability of matrix array transducers to produce an ARFI excitation, this simulation analysis lays the groundwork for mechanisms to improve 3-D SWEI.

## V. Conclusion

Reflected waves violate the assumption of a single direction of propagation made by TOF SWS reconstruction methods, leading to artifacts in SWS images. Directional filters reject these reflected wave artifacts, leading to improved image reconstructions that have reduced variance in bias and higher CNR. Increasing the dimensionality of the directional filter rejects more of these shear wave artifacts, allowing for the reconstruction of more accurate SWS images. The improvement in image quality depends on the severity of the artifact, which is a function of both the lesion characteristics and the geometry of the excitation beam. The 3-D filtering shows significant improvements in image quality over 2-D filtering, and because it can be performed on data obtained with a 1-D array at moderate computational cost, it would benefit most existing SWEI imaging implementations. While 4-D-directional filtering shows modest improvements over 2-D and 3-D filtering, the realistic implementation of these filters is limited by computational overhead of large data sets and the higher cost of the necessary matrix array transducers. If a matrix array is available, 4-D-directional filtering will provide the greatest rejection of shear wave reflection artifacts and allow for an SWS volume to be reconstructed.

## Figures and Tables

**TABLE I:** Gaussian ARFI Excitation Parameters as Applied in (2). All of the Excitations Had *z*_0_ = 21 mm, *σ_z_* = 4.25 mm, and *T*_ON_ = 80 *μ*s. The Excitations Demonstrate Different Degrees of Spatial Asymmetry in the Lateral–Elevation Extent of the Acoustic Radiation Force Source That Influences the Shear Wave Frequency Content

	Excitation 1	Excitation 2	Excitation 3

*σ_x_*	0.21 mm	0.21 mm	0.43 mm
*σ_y_*	0.21 mm	0.85 mm	0.43 mm
*A*	0.11 dyn	0.11 dyn	0.05 dyn

**TABLE II:** Fourier Dimensions of Directional Filters

Filter Dimensionality	Spatio-Temporal Dimensions	Frequency Dimensions

2D	*x, t*	*k_x_*, *ω*
3D	*x, z, t*	*k_x_*, *k_z_*, *ω*
4D	*x, y, z, t*	*k_x_*, *k_y_*, *k_z_*, *ω*

## References

[1] Sarvazyan AP, Rudenko OV, Swanson SD, Fowlkes JB, Emelianov S (1998). Shear wave elasticity imaging: A new ultrasonic technology of medical diagnostics. Ultrasound Med Biol.

[2] Nightingale K, McAleavey S, Trahey G (2003). Shear-wave generation using acoustic radiation force: *In vivo* and *ex vivo* results. Ultrasound Med Biol.

[3] Bercoff J, Tanter M, Fink M (2004). Supersonic shear imaging: A new technique for soft tissue elasticity mapping. IEEE Trans Ultrason, Ferroelectr, Freq Control.

[4] Sandrin L, Tanter M, Gennisson JL, Catheline S, Fink M (2002). Shear elasticity probe for soft tissues with 1-D transient elastography. IEEE Trans Ultrason, Ferroelectr, Freq Control.

[5] Sandrin L, Tanter M, Catheline S, Fink M (2002). Shear modulus imaging with 2-D transient elastography. IEEE Trans Ultrason, Ferroelectr, Freq Control.

[6] Palmeri ML, Wang MH, Dahl JJ, Frinkley KD, Nightingale KR (2008). Quantifying hepatic shear modulus *in vivo* using acoustic radiation force. Ultrasound Med Biol.

[7] Doherty JR, Trahey GE, Nightingale KR, Palmeri ML (2013). Acoustic radiation force elasticity imaging in diagnostic ultrasound. IEEE Trans Ultrason, Ferroelectr, Freq Control.

[8] Rouze NC, Wang MH, Palmeri ML, Nightingale KR (2012). Parameters affecting the resolution and accuracy of 2-D quantitative shear wave images. IEEE Trans Ultrason, Ferroelectr, Freq Control.

[9] Tanter M (2008). Quantitative assessment of breast lesion viscoelasticity: Initial clinical results using supersonic shear imaging. Ultrasound Med Biol.

[10] Deffieux T, Gennisson JL, Bercoff J, Tanter M (2011). On the effects of reflected waves in transient shear wave elastography. IEEE Trans Ultrason, Ferroelectr, Freq Control.

[11] Song P, Zhao H, Manduca A, Urban MW, Greenleaf JF, Chen S (2012). Comb-push ultrasound shear elastography (CUSE): A novel method for two-dimensional shear elasticity imaging of soft tissues. IEEE Trans Med Imag.

[12] Song P, Urban MW, Manduca A, Zhao H, Greenleaf JF, Chen S (2013). Comb-push ultrasound shear elastography (CUSE) with various ultrasound push beams. IEEE Trans Med Imag.

[13] Song P, Manduca A, Zhao H, Urban MW, Greenleaf JF, Chen S (2014). Fast shear compounding using robust 2-D shear wave speed calculation and multi-directional filtering. Ultrasound Med Biol.

[14] Gennisson JL (2015). 4-D ultrafast shear-wave imaging. IEEE Trans Ultrason, Ferroelectr, Freq Control.

[15] Palmeri ML, Sharma AC, Bouchard RR, Nightingale RW, Nightingale KR (2005). A finite-element method model of soft tissue response to impulsive acoustic radiation force. IEEE Trans Ultrason, Ferroelectr, Freq Control.

[16] Wang M, Byram B, Palmeri M, Rouze N, Nightingale K (2013). On the precision of time-of-flight shear wave speed estimation in homogeneous soft solids: Initial results using a matrix array transducer. IEEE Trans Ultrason, Ferroelectr, Freq Control.

[17] Wang M, Byram B, Palmeri M, Rouze N, Nightingale K (2013). Imaging transverse isotropic properties of muscle by monitoring acoustic radiation force induced shear waves using a 2-D matrix ultrasound array. IEEE Trans Med Imag.

[18] Palmeri ML, McAleavey SA, Fong KL, Trahey GE, Nightingale KR (2006). Dynamic mechanical response of elastic spherical inclusions to impulsive acoustic radiation force excitation. IEEE Trans Ultrason, Ferroelectr, Freq Control.

[19] Manduca A, Lake DS, Kruse SA, Ehman RL (2003). Spatio-temporal directional filtering for improved inversion of MR elastography images. Med Image Anal.

[20] Slawinski MA (2010). Waves and Rays in Elastic Continua.

[21] Zhao H (2011). Bias observed in time-of-flight shear wave speed measurements using radiation force of a focused ultrasound beam. Ultrasound Med Biol.

[22] Palmeri ML, McAleavey SA, Trahey GE, Nightingale KR (2006). Ultrasonic tracking of acoustic radiation force-induced displacements in homogeneous media. IEEE Trans Ultrason, Ferroelectr, Freq Control.

[23] Rouze NC, Wang MH, Palmeri ML, Nightingale KR (2010). Robust estimation of time-of-flight shear wave speed using a radon sum transformation. IEEE Trans Ultrason, Ferroelectr, Freq Control.

[24] Smith SW, Wagner RF (1984). Ultrasound speckle size and lesion signal to noise ratio: Verification of theory. Ultrason Imag.

[25] Deffieux T, Gennisson JL, Larrat B, Fink M, Tanter M (2012). The variance of quantitative estimates in shear wave imaging: Theory and experiments. IEEE Trans Ultrason, Ferroelectr, Freq Control.

[26] Palmeri ML, Deng Y, Rouze NC, Nightingale KR (2014). Dependence of shear wave spectral content on acoustic radiation force excitation duration and spatial beamwidth. Proc IEEE Int Ultrason Symp (IUS).

[27] Rouze NC, Palmeri ML, Nightingale KR (2015). An analytic, Fourier domain description of shear wave propagation in a viscoelastic medium using asymmetric Gaussian sources. J Acoust Soc Amer.

